# A mixed methods protocol to implement universal firearm injury risk screening and intervention among youth and adults in emergency departments across a large US health system

**DOI:** 10.1186/s43058-022-00371-6

**Published:** 2022-11-24

**Authors:** Chethan Sathya, Laura Harrison, Katherine Dauber, Sandeep Kapoor

**Affiliations:** 1 Division of Pediatric Surgery, Cohen Children’s Medical Center, Northwell Health, New Hyde Park, NY USA; 2 Donald and Barbara Zucker School of Medicine at Hofstra/Northwell, Hempstead, NY USA; 3 Center for Gun Violence Prevention, Northwell Health, New Hyde Park, NY USA; 4 Addiction Services, Emergency Medicine Service Line, Northwell Health, 1111 Marcus, Suite M15, New Hyde Park, NY 1104211042 USA; 5 Institute for Health System Science, Feinstein Institutes, Northwell Health, New Hyde Park, USA

## Abstract

**Background:**

Firearm injury is a leading cause of preventable death in the USA. Healthcare providers are uniquely poised to focus on firearm safety and injury prevention from an apolitical harm reduction lens; however, few providers and healthcare settings incorporate firearm injury prevention strategies into usual care. We outline the first protocol to determine *how* to implement universal Firearm Injury and Mortality Prevention (FIMP) strategies that identify and address firearm access and violence risk in healthcare settings as part of routine care using the Consolidated Framework for Implementation Research (CFIR) to inform implementation and evaluation.

**Methods:**

The components of our FIMP strategy, including universal screening, intervention for patients at risk, and resources, will be developed from existing evidence-based strategies for firearm access and violence risk (*intervention characteristics*). The implementation process will include components of Screening, Brief Intervention, and Referral to Treatment (SBIRT) for substance use, adapted to FIMP (*implementation process*). To engage stakeholders, harmonize language, and garner support, an Executive Advisory Board (EAB) will be formed, consisting of the site- and system-level stakeholders (*inner setting*) and community stakeholders, including influential figures such as local religious and spiritual leaders, individuals with lived experience, and community-based organizations (*outer setting*). Pre-implementation surveys will identify the *characteristics of individuals* and guide the development of education prior to implementation. Patient-level screening data will be analyzed to identify the risk factors, implementation will be evaluated using mixed methods, and a limited-efficacy study will evaluate whether strategies were successful in driving behavior change.

**Discussion:**

This study protocol has breakthrough and methodological innovations, by addressing FIMP as part of usual care to directly mitigate firearm injury risk among youth, adults, and household members (e.g., children) and by using rigorous methods to inform healthcare industry implementation of FIMP strategies. The expected outcomes of this study protocol will provide a solid basis for larger-scale dissemination and evaluation of implementation, effectiveness, and usability across broader pediatric and adult healthcare settings. This project will advance the implementation science and have a positive impact on the health of our patients and communities by preventing firearm injury and mortality and shifting the paradigm to view FIMP through a public health lens.

Contributions to the literature

- This project has breakthrough and methodologic innovations, by addressing FIMP as part of usual care to mitigate firearm injury risk among youth, adults, and household members and using rigorous methods to inform healthcare implementation of FIMP strategies.
- The expected outcomes of this project will provide a basis for larger-scale dissemination and evaluation of implementation, effectiveness, and usability across broader pediatric and adult healthcare settings.
- This project will advance the implementation science and have a positive impact on the health of our patients and communities by preventing firearm injury and shifting the paradigm to view FIMP through a public health lens.

## Background

Firearm injury is a leading cause of preventable death in the USA and the second leading cause of injury death after motor vehicle collisions [1–4]. With a recent surge in firearm purchases among Americans during the COVID-19 pandemic, increasing social isolation and inequity, 2020 was the deadliest year on record for gun violence in the USA [5, 6]. The homicide rate increased by 35% from 2019 to 2020, with increasing disparities by race, ethnicity, and poverty level [7], and firearm injuries overtook motor vehicle crashes as the leading cause of death in children and adolescents [8]. Firearm-related homicide is highest for adolescents and young adults, with higher rates in Black/African American males, while firearm-related suicide is highest in middle-aged and older White males [9]. Healthcare environments serve as crucial venues to support the cries of our communities, and emergency departments (EDs) serve as a safety net for the unmet needs of our most vulnerable patients and are often the only touchpoint with healthcare. In the context of firearm injury prevention, healthcare providers are uniquely positioned to focus on firearm safety and injury prevention from an apolitical harm reduction standpoint [10]. In order to effectively apply a public health approach to mitigate firearm injury risk, it is essential to implement preventative strategies to broadly identify the risk with subsequent tailored interventions [10].

Evidence-based firearm injury and mortality prevention (FIMP) strategies exist, at primary (pre-injury), secondary (injury), and tertiary (post-injury) prevention levels [10]. Traditionally, the healthcare system provides care at the point of injury, but there is ample opportunity to incorporate preventative strategies both pre- and post-injury. Pre-injury strategies include screening, firearm safety counseling, gun lock distribution, motivational interviewing, and community resources such as violence interrupters; post-injury strategies include access to mental health and community support [11–18]. Despite consensus on care and research priorities for addressing FIMP in the healthcare setting, there are gaps in knowledge limiting our understanding of the facilitators and barriers to programmatic implementation [10, 19]. Consequently, though there is a widespread acceptance of the need for screening/counseling to improve firearm safety exists among both clinical team members and patients, it is done infrequently [20–22]. Humanistic, non-judgmental verbiage related to firearm injury screening and counseling is far from commonplace in most health professionals’ skillsets [21]. Prior limited FIMP studies have focused on outpatient settings or used targeted, not universal screening which can exclude at-risk patients presenting with an unrelated issue (missed opportunities), influencing understanding of the risk in diverse cohorts, and creating a stigmatized environment (barriers), further limiting implementation [11–18, 21].

There is a critical need to implement a system-level FIMP strategy to shift the paradigm to view this as a public health issue with modifiable risk factors the healthcare industry can address as part of usual care [22]. Determining how to implement FIMP strategies is central to disseminating preventative strategies and providing infrastructure for downstream research.

*Universal screening *has the potential to maximize reach and create a stigma-free environment. Our approach to FIMP will be based on a foundation of universal screening to normalize the conversation as part of usual care for both the healthcare team and patients. At our institution, we successfully implemented a “We Ask Everyone” approach with Screening, Brief Intervention, and Referral to Treatment (SBIRT) for alcohol and drug use [23, 24]. There has been a success with universal screening for other healthcare issues to decrease stigma and increase testing, such as with HIV [25]. Universal screening, combined with education for healthcare professionals, has widened our reach and reduced the stigma surrounding addressing substance use in the healthcare setting [26, 27]. Previous work evaluating interventions similar to SBIRT for violence prevention (SafERteens) and firearm safety counseling (i.e., safety check) have shown positive results in reducing the risk of future injury and increasing safe firearm storage behaviors [9, 12, 19, 20, 28]. We will apply the framework used for SBIRT for substance use to FIMP, with synergistic goals of maximizing reach to all patients and destigmatizing firearm injury risk.

We outline the first published research protocol to develop, implement, and evaluate the feasibility of evidence-based universal screening and intervention among youth and adults at risk of firearm injury across EDs in a large US health system. This is the first protocol to comprehensively outline an approach to educate clinical team members and implement evidence-based screening and intervention for both firearm access and violence risk. We will utilize principles of implementation science to determine and evaluate *how* to implement FIMP strategies into the healthcare setting allowing for the effective dissemination of preventative strategies. The Consolidated Framework for Implementation Research (CFIR) [29, 30] will provide the pre-implementation framework for identifying the barriers and facilitators to developing and implementing our FIMP strategy. CFIR is intended to provide a pragmatic structure to translate evidence-based interventions into meaningful patient care outcomes by identifying constructs within the following domains: (1) *intervention characteristics*, (2) *outer setting*, (3) *inner setting*, (4) *characteristics of individuals*, and (5) *process* [29]. To address the limitations of CFIR, we will further incorporate Expert Recommendations for Implementing Change (ERIC), including “identify and prepare champions,” “assess for readiness and identify barriers and facilitators,” “identify early adopters,” and “conduct educational meetings.” [31].

### Objectives, aims, and hypotheses

One of our objectives is to study *how* to successfully implement FIMP strategies across healthcare settings using CFIR. The long-term goal of our implementation research is to inform industry practices to efficiently implement strategies for screening and intervention among patients at risk of firearm injury.

- Aim 1: Use mixed methods to inform the development and implementation of evidence-based universal screening for firearm injury risk, consisting of screening for access and violence risk, and brief FIMP intervention (motivational interviewing, education, resources) at the point of care among at-risk youth and adults in pilot EDs
- Aim 2: Pilot the FIMP screening and intervention strategy at 3 ED sites and evaluate the feasibility of *implementation* in the following domains: (1) *acceptability*, (2) *demand* for the intervention and resources, (3) success of *implementation*, (4) *practicality* of the intervention, (5) *adaptation* of the intervention to future iterations, (6) *integration* of the intervention in pilot setting, (7) *expansion* of the intervention to additional settings, and (8) *limited-efficacy testing* through which we evaluate *outcomes*, including safe firearm storage practices, behavior changes, and community resource use among patients receiving an intervention [32]

We hypothesize that by identifying the barriers and facilitators to implementation and the knowledge gaps among clinical team members and patients, we will design an effective education that will increase knowledge and comfort with FIMP and develop strategies to effectively implement universal FIMP screening and targeted interventions for adolescent and adult patients at risk in ED settings. By iteratively incorporating feedback from team members, stakeholders, and patients and ongoing programmatic evaluation, we will successfully implement screening and intervention in three pilot EDs and gain a better understanding of facilitators and barriers to implementation, patient/provider experience, characteristics associated with firearm injury and mortality risk, and intervention efficacy and outcomes.

## Methods

A mixed methods design will be used to develop, implement, and evaluate an evidence-based universal screening tool and tailored intervention to address firearm injury and mortality risk among our ED patients as part of usual care. Prior to implementation, we will also use CFIR to identify the facilitators and barriers and guide our FIMP implementation strategy [29].

### Development of evidence-based FIMP strategies

#### Screening

Evidence-based screening for firearm injury risk will include asking patients about firearm access and predictors of future firearm violence. We will adapt these questions into an innovative screening tool and incorporate this into the electronic health record (EHR). Firearm access is associated with increased firearm-related homicide, suicide, and unintentional injury and mortality [9, 33], suggesting screening for firearm access within or outside the home (i.e., in the community, friend group, or other households) is an essential component of ascertaining firearm injury risk. The SaFETy score, inclusive of predictors of violence risk, is the only existing validated clinical screening tool for firearm injury risk in an ED setting [11]. Though only validated in youth 14–24, we will extrapolate the use of the tool to a broader ED patient population [11]. The tool includes four questions (serious fighting, friend weapon carrying, community environment, and firearm threats) to stratify future firearm violence risk. Adolescents and adults ages 12 and older will be asked about firearm *access* (not ownership or registration) within or outside the household. Violence risk will be assessed using the SaFETy score [11] for adolescents ages 12–17 and with a one-question prescreen based on the SaFETy [11] score for adults 18 and older.

#### Screening process

**Fig. 1:**
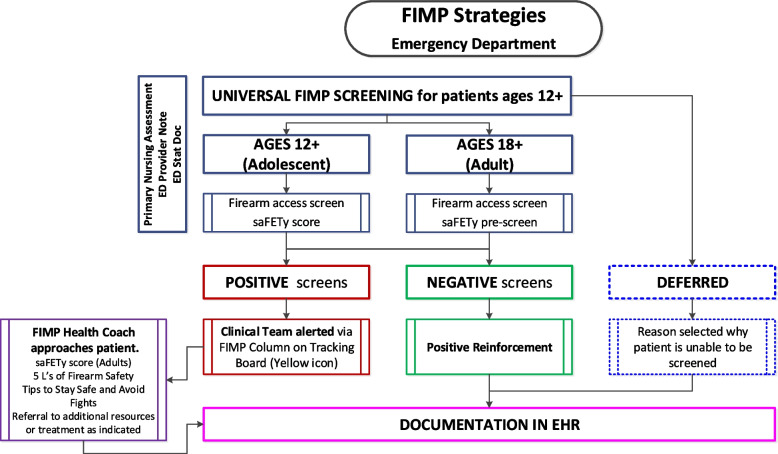
ED workflow for firearm injury risk screening and intervention

Our work developing and implementing SBIRT screening for substance use has demonstrated the importance of the “We Ask Everyone” approach to universal screening [24, 26]. We will maintain this approach in our FIMP strategy by screening all ED patients ages 12 and older for firearm injury risk, with exceptions for patients who are too sick or refuse (Fig. 1). Screening questions will be available in the EHR to physicians, nurses, and social workers, and an automatic icon will alert the clinical team of a “positive” screening, indicating the patient should be approached by a FIMP health coach. Additional risk factors including substance use, depression, suicidality, homicide risk, and abuse [34] are currently assessed as part of usual care and will be incorporated into the conversation with the patient.

### Brief intervention for patients at risk

There is evidence to support existing strategies that address both firearm access and violence risk. For access, safe storage counseling augmented by gun safety device provision has been shown to be an effective means of changing safe storage behaviors [12–15]. ED- and hospital-based violence intervention strategies have been shown to reduce firearm violence in adolescents and young adults [18, 35–38]. Our FIMP intervention will incorporate the aspects of previously successful strategies in both access and violence risk domains.

**Fig. 2:**
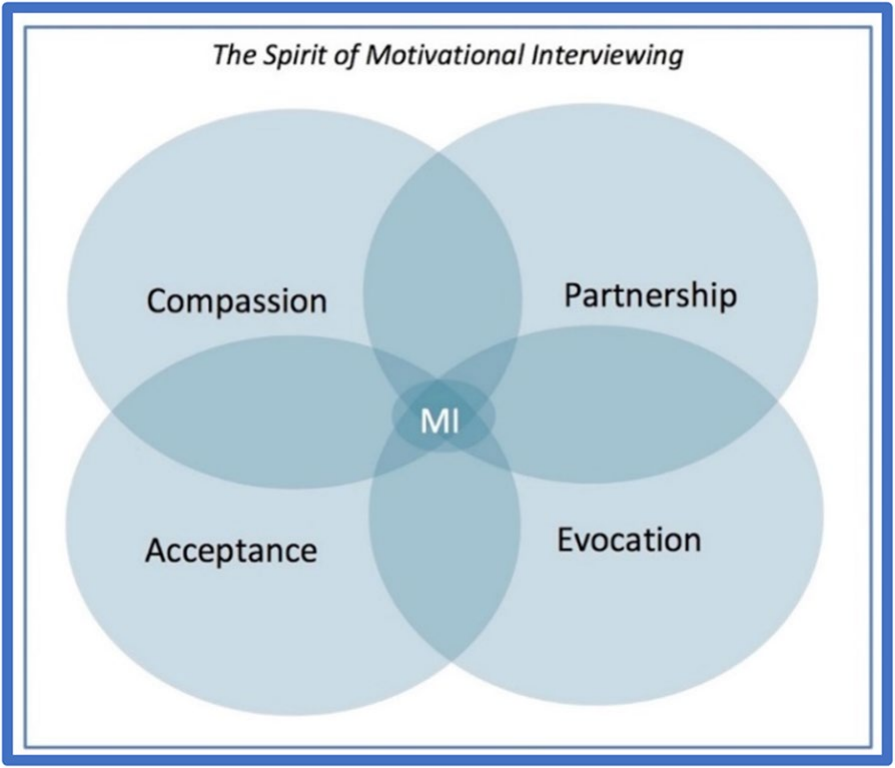
The spirit of motivational interviewing

FIMP brief interventions will be conducted using Brief Negotiated Interview (BNI) based on Prochaska and DiClemente’s Theoretical Model of Behavioral Change and in the spirit of motivational interviewing (MI), adapted from SBIRT for substance use [39, 40]. In response to a “positive” screen, a FIMP health coach will approach the patient for a Brief Negotiated Interview (BNI), modified from addressing substance use [41–46]. MI includes a focus on compassion, acceptance, evocation, and partnership (Fig. 2) and uses the empathy microskills of reflection, legitimation, and exploration [47], to allow patients to reflect on their own insights and motivations to contemplate changes. The brief intervention should take 10–15 min.

There are four steps to the BNI: (1) *raise the subject*, (2) *provide feedback*, (3) *enhance motivation*, and (4) *plan*. To *raise the subject*, health coaches will introduce the topic of firearm injury prevention to patients, mentioning the questions they were asked earlier in their visit and normalizing the topic by explaining “We Ask Everyone.” They will review the responses to access and violence risk questions, ask the full SaFETy score for adult patients, and review the patient’s responses to other health risk factor questions, including substance use and depression.

To *provide feedback *for patients with firearm access (similar to “safety check”) [12], health coaches will offer information on the 5 L’s of Firearm Safety (Locked, Loaded, Little children, feeling Low, and Learned owner) [16, 17, 30]. For violence risk, health coaches will suggest tips to stay safe and avoid fights and other components of SafERteens, an intervention that has been demonstrated to reduce future violence risk [48, 49]. Health coaches will use a readiness ruler to *enhance motivation*, allowing the patient to identify their readiness for change, and to reiterate *their* motivations for change. To *plan*, health coaches will be trained to support the patient’s will to incorporate any strategies for harm reduction or injury prevention, in addition to partnering with the patient to devise a collaborative plan for the next steps that are representative of the *patient’s* safety goals.

#### Resources

Based on screening responses, stratified risk level, and motivation for change, patients will be offered and connected to appropriate supports that will best meet their needs. For patients who have firearm *access*, resources will be provided for safe storage such as gun locks and information on purchasing gun lockboxes/safes. For those whose safety goal is to dispose of a firearm and/or ammunition, information will be provided on local gun buyback events and options for safe disposal of ammunition, including calling ahead to a local police precinct, gun stores, shooting ranges, and hazardous/special materials disposal events. For the “Low” aspect of the 5 L’s of Firearm Safety, information will be provided on the health system and community-based behavioral health providers appropriate for any household member who may benefit [50, 51]. For “Learned Owner,” information will be provided for virtual and local in-person firearm safety classes. For patients whose SaFETy score indicates *violence risk*, a hospital-based intervention program (HVIP) will be activated, whereby a member of a community violence intervention program or credible messenger can either meet with the patient in person or speak to the patient by phone during their ED visit to establish a relationship [52, 53]. Information will also be provided on local community-based organizations and mentorship programs. For those with mental health or substance use treatment needs, a referral to the appropriate level of treatment will be offered to a state-licensed mental health or substance use treatment provider [50, 54–56].

### Pre-implementation

Pre-implementation surveys will be conducted with site-level and system-level stakeholders, with Likert-scale and narrative questions. Feedback obtained will inform educational development and delivery, messaging, and implementation workflows. Pre-implementation surveys will address CFIR domains [29] to guide the next steps and facilitate successful implementation.

#### Patient and Public Involvement

We will engage key stakeholders, including members of the community and our organization, as well as patients, to join our Executive Advisory Board (EAB). The EAB will include system-level stakeholders from the Northwell Emergency Medicine Service Line, the Northwell Health Center for Gun Violence Prevention, and the departments of Medicine, Trauma, Pediatrics, Community Relations, and Legal; site-level stakeholders from each pilot site (*inner setting*); and community stakeholders, including influential figures such as local religious and spiritual leaders, individuals and families of those who have suffered firearm violence, school-based professionals, and community-based organizations (*outer setting*) [29]. The overarching goal of the EAB will be to partner with the centralized study team to build up a network of services and inform system-level messaging to facilitate the acceptability of protocols.

#### Stakeholder and pilot site engagement

Pre-implementation surveys will be administered during multidisciplinary exploratory meetings at each pilot site. Based on the feedback from stakeholders, we will develop a pre-implementation checklist, including key contingencies that need to be resolved prior to go-live. The checklist will include (1) delegation of duties to clinical team members, (2) development of resource documents highlighting options for post-ED follow-up, and (3) identification of champions [31]. Any motivated team member can serve as a champion; their role will be to disseminate messaging around the FIMP initiative, assist in motivating team members to complete surveys, participate in additional training to be able to better inform and empower team members, and assist team members with adding the firearm injury risk screening to their electronic health record (EHR) profile.

#### Implementation surveys

Before implementation, we will survey clinical, non-clinical, and community stakeholders to assess attitudes, knowledge, perceived skills, and anticipated barriers and facilitators to addressing FIMP in the ED. Our pre-implementation assessment will address the *characteristics of individuals* domain of CFIR, including *knowledge and beliefs*regarding the proposed FIMP strategies [29]. Surveys will also identify staff attitudes towards process *intervention characteristics*, including the *intervention source*, *evidence strength and quality*, *relative advantage*, and *adaptability* [29]. Pre- and post-implementation surveys will utilize open-ended and Likert scale questions and semi-structured interviews. Likert scale questions will be analyzed using paired-samples *t*-tests, and the results will be stratified by cohort (physicians, nurses, social workers, etc.). Feedback will inform the development of our educational curricula and integration of FIMP strategies into ED clinical workflow.

#### Education

Two levels of education will be developed: one level for team members who will only be screening for firearm injury risk, and the second level for team members who will be trained as FIMP health coaches to meet with patients who screen positive for firearm injury risk. Education will be iteratively developed and refined based on pre-implementation surveys, site champion meetings, and survey evaluations of educational sessions.

Firearm injury prevention is not routinely approached in healthcare settings, resulting in a lack of awareness, expertise, and comfort among healthcare professionals and patients [21]. To normalize the conversation and ensure a positive *inner setting*prior to pilot go-live, the entire interprofessional healthcare team will receive foundational education. Based on lessons learned from Northwell SBIRT [24, 26], this is a critical step to foster engagement, comfort, and motivation. All frontline team members who will be completing FIMP screening at pilot sites will complete a 20-min asynchronous online training module prior to go-live at their site. Learning objectives will include exploring firearm injury as a public health issue, exploring what the FIMP initiative IS and IS NOT, and reviewing the screening process. The material will also be presented live virtually and/or in person, capitalizing on faculty meetings, resident training sessions, grand rounds, nursing huddles, and other educational opportunities.

Team members who will serve as FIMP health coaches and meet with patients with a positive screening will attend workshops delivered in person with a simultaneous virtual option. The content will focus on the knowledge and skills needed to deliver a brief intervention and resources to patients who screen positive for firearm injury risk through access and/or violence risk pathways, including anatomy of a firearm, motivational interviewing, how to conduct a brief intervention for FIMP, resources available, and where to find them. Participants will be provided with a learner guide including patient-facing handouts with information and resources, frequently asked questions, workflow, and role-play cases. The broad approach to offer education to all interested parties will ensure (1) wide availability of FIMP services for patients who screen positive, (2) harmonization of language and approach, and (3) communal comfort with supporting patients at risk. We will use pre- and post-session surveys to evaluate the achievement of learning objectives and inform future iterations.

### Implementation and evaluation

We will pilot the FIMP screening and intervention strategies at three pilot EDs.

#### Evaluation

Service delivery including reach/adoption [51] will be evaluated using the following metrics: (1) percent of eligible patients screened or with a documented reason for no screening, (2) percent of screened patients with “positive” screening, (3) patients with positive screening who received a brief intervention and resources, (4) patients with positive screening unable to receive services from a FIMP Health Coach, (5) saFETy scores and reported firearm safety behaviors, and (6) referrals to community resources and treatment providers. Screening data will be evaluated to identify the characteristics of the cohorts at highest risk within our patient population and to identify the highest risk days/times to inform future downstream services, targeting of interventions, and staffing. Data will be analyzed to begin to answer some of the questions identified as high priority for FIMP research in emergency medicine, including (a) settings in which FIMP screening and intervention are feasible and acceptable, (b) whether screening should be universal, (c) the types of research approaches that would improve the study of firearm injury, and (d) identification of outcome measures for firearm injury research [19].

#### Limited-efficacy testing

Patient experience, attitudes, and behavior change around firearm safety will be assessed using a quasi-experimental interrupted time-series approach for a subset of patients who receive brief intervention services during their ED visit. Contact information will be collected for follow-up by FIMP health coaches at 1 week and at 3 months to ask about patient experience, change in attitudes, and firearm safety behavior, compared to during the ED visit.

#### Data analysis

Pre- and post-survey data for implementation and educational sessions will be entered into REDCap and exported to IBM SPSS Statistics for analysis [57, 58]. Quantitative Likert scale questions will be analyzed using paired samples *t*-tests, stratified by site and staff role/cohort. Qualitative data will be analyzed using a step-by-step thematic analysis without a pre-specified coding frame [59]. FIMP screening data will be exported from the EHR into reports that include visit data (date/time, chief complaint, diagnosis), demographic data (age, gender, race, ethnicity, primary language), and substance use and mental health screening data. Patient-level data will allow an analysis of firearm injury risk levels by other demographic and health characteristics. Logistic regression will be used to analyze the firearm injury screening results (positive/negative) by demographic factors, including gender, age group, race, ethnicity, primary language, and whether the chief complaint was related to firearm injury, mental health, or substance use.

#### Dissemination of findings

The results will be disseminated through conference presentations, peer-reviewed publications, and through the Gun Violence Prevention Learning Collaborative for Health Systems and Hospitals, which is hosted by Northwell Health and has over 400 participants from hospitals across 35 states.

## Discussion

This study has a major potential impact toward improving public health, by addressing both firearm access and firearm violence as part of usual care to directly mitigate firearm injury risk among youth, adults, and household members (e.g., children) and by using rigorous methods to inform healthcare industry implementation of FIMP strategies. The universal screening will serve to normalize and de-stigmatize the conversation around firearm injury risk as part of usual care for both the healthcare team and patients, will provide every patient with the opportunity to discuss firearm injury risk with their healthcare team, and will demonstrate the characteristics of ED patients at risk of firearm injury.

This study protocol is also scientifically significant for its potential to contribute to the field of implementation science, at a systems level. Current gaps in the implementation literature include a lack of clarity on how to move beyond studied barriers to implementation and how to move beyond evidence-based practice to standard care and generalizable knowledge. Our aims will add to the literature, as we intend to use the CFIR to identify facilitators, not just barriers, to implementation that will serve to inform dissemination of FIMP strategies into usual care. By evaluating the feasibility and demonstrating a successful model of FIMP strategy implementation within a large integrated health system, we will facilitate a shift in the paradigm to view firearm injury as a public health issue. This study protocol and its results will be valuable to the healthcare industry and serve as motivation and support for health systems nationally to implement FIMP strategies. Additionally, universal screening and downstream service delivery data will generate insights into the associations between patient-level characteristics (demographics, risk factors) and firearm access and injury risk. Follow-up data will capture the patient experience and allow us to evaluate the efficacy of implemented FIMP strategy, from the patient’s perspective, a missing domain in the current literature.

Several limitations do exist. This protocol is based on the screening tools and intervention components that have only been validated and studied in certain populations. For example, the SaFETy score was initially validated in substance-using youth ages 14–24 who presented to the ED [11]. The SafERteens intervention was tested in the same demographic [38]. Interventions for firearm access have been tested mostly in ambulatory care settings, with parents of pediatric patients and geriatric patients [12–18]. However, this study will serve to establish the feasibility of using these interventions in a broader ED population, ages 12 and older. With regard to effectiveness, the limited-efficacy study will not include a control group, limiting the strength of the evidence that will be obtained. While the study will evaluate the behavior change, there will be no comparison group to account for other societal changes, education, and healthcare interventions outside of the FIMP strategies provided. However, this study will provide data to help inform a larger-scale prospective study that includes a control group. Despite these limitations, this is the first study protocol aiming to identify and overcome the barriers to the implementation of evidence-based FIMP strategies across a large health system, a knowledge gap that must be filled in order to improve public health across at-risk communities.

The expected outcomes of this study will provide a solid basis for larger-scale dissemination and evaluation of FIMP implementation, effectiveness, and usability across broader healthcare settings. This project will advance the implementation science and have a positive impact on the health of our patients and communities by preventing firearm injury and mortality and shifting the paradigm to view FIMP through a public health lens.

## Data Availability

Pertinent data generated or analyzed as a result of this study protocol in the future will be made available in publications, and some portions could be made available from the corresponding author upon reasonable request.
